# INTER-ACT: prevention of pregnancy complications through an e-health driven interpregnancy lifestyle intervention – study protocol of a multicentre randomised controlled trial

**DOI:** 10.1186/s12884-017-1336-2

**Published:** 2017-05-26

**Authors:** Annick Bogaerts, Lieveke Ameye, Margriet Bijlholt, Kelly Amuli, Dorine Heynickx, Roland Devlieger

**Affiliations:** 1 Department of Development and Regeneration, KU Leuven, Herestraat 49, 3000 Leuven, Belgium; 2 Research Unit Healthy Living, Faculty of Health and Social Work, University College Limburg-Leuven, Wetenschapspark 21, 3590 Diepenbeek, Belgium; 3 Department of Nursing and Midwifery, CRIC Centre for Research & Innovation in Care, University of Antwerp, Universiteitsplein 1, 2610 Wilrijk, Belgium; 4 Department of Obstetrics, Gynecology and Reproduction, St-Augustinus Hospital Wilrijk, Oosterveldlaan 24, 2610 Wilrijk, Belgium; 5 Department of Obstetrics and Gynecology, University Hospital Leuven, Herestraat 49, 3000 Leuven, Belgium

**Keywords:** RCT, Interpregnancy lifestyle intervention, Maternal obesity, Excessive gestational weight gain, Pregnancy-induced hypertension, Gestational diabetes, Caesarean section, Large-for-gestational-age

## Abstract

**Background:**

Excessive maternal pre-pregnancy and gestational weight gain are related to pregnancy- and birth outcomes. The interpregnancy time window offers a unique opportunity to intervene in order to acquire a healthy lifestyle before the start of a new pregnancy.

**Methods:**

INTER-ACT is an e-health driven multicentre randomised controlled intervention trial targeting women at high risk of pregnancy- and birth related complications. Eligible women are recruited for the study at day 2 or 3 postpartum. At week 6 postpartum, participants are randomised into the intervention or control arm of the study. The intervention focuses on weight, diet, physical activity and mental well-being, and comprises face-to-face coaching, in which behavioural change techniques are central, and use of a mobile application, which is Bluetooth-connected to a weighing scale and activity tracker. The intervention is rolled out postpartum (4 coaching sessions between week 6 and month 6) and in a new pregnancy (3 coaching sessions, one in each trimester of pregnancy); the mobile app is used throughout the two intervention phases. Data collection includes data from the medical record of the participants (pregnancy outcomes and medical history), anthropometric data (height, weight, waist- and hip circumferences, skinfold thickness and body composition by bio-electrical impedance analysis), data from the mobile app (physical activity and weight; intervention group only) and questionnaires (socio-demographics, breastfeeding, food intake, physical activity, lifestyle, psychosocial factors and process evaluation). Medical record data are collected at inclusion and at delivery of the subsequent pregnancy. All other data are collected at week 6 and month 6 postpartum and every subsequent 6 months until a new pregnancy, and in every trimester in the new pregnancy. Primary outcome is the composite endpoint score of pregnancy-induced hypertension, gestational diabetes mellitus, caesarean section, and large-for-gestational-age infant in the subsequent pregnancy.

**Discussion:**

INTER-ACT is a unique randomised controlled lifestyle intervention trial in its implementation between pregnancies and during the subsequent pregnancy, with an e-health driven approach.

**Trial registration:**

ClinicalTrials.gov Identifier: NCT02989142. Registered August 2016.

## Background

Maternal pre-pregnancy weight is related to pregnancy- and birth outcomes. An excessive weight before conception increases the risk for pregnancy- and birth related complications such as gestational diabetes mellitus (GDM), pregnancy-induced hypertension (PIH), caesarean section (CS), or a large-for-gestational age (LGA) infant [1–4]. Besides, excessive gestational weight gain (EGWG), i.e. a gestational weight gain (GWG) higher than the recommended GWG by the Institute Of Medicine (IOM) [5], is also associated with these perinatal complications [6–8]. Both the pre-pregnancy BMI and gestational weight gain (GWG) are thus considerable risk factors of pregnancy and birth complications. Between 2009 and 2014, the Flanders Study centre Perinatal Epidemiology (SPE) collected data of almost 400.000 singleton pregnancies in Flanders, Belgium, on the combined association of GWG and pre-pregnancy BMI with the prevalence of the composite outcome of pregnancy and birth complications (i.e. at least one of four perinatal outcomes PIH, GDM, CS and/or LGA infant). The prevalence of the composite perinatal outcome was 26% in women with a normal BMI and an adequate GWG; 34% in women with a normal BMI but with an excessive GWG; and 66% in women with class III obesity (BMI ≥ 40) and excessive GWG [SPE, 2016 in progress]. A Norwegian study reported similar findings. Haugen et al. [9] found that normal weight and overweight women with EGWG had an increased risk for PIH, preeclampsia, CS, high birth weight (>4500 g) and LGA infant. Moreover, women of all BMI classes and with EGWG, except underweight women, had an increased risk of more than 2 kg postpartum weight retention (PPWR) at 18 months postpartum [9]. Conversely, a more recent study from Xiong et al. reported an increased likelihood of CS in underweight or normal weight women with EGWG compared to overweight or obese women EGWG [10]. Thus, these results highlight the importance of adequate GWG in all BMI groups.

Based on the guidelines of the Institute of Medicine (IOM) [5], one in three women in Flanders, Belgium, has excessive GWG: 25% in normal pre-pregnancy BMI, 58% in pre-pregnancy overweight, and 54% in pre-pregnancy obesity [11]. Half of the women with excessive GWG do not return to their pre-pregnancy weight after delivery, resulting in a doubled risk for pregnancy- and birth related complications in the next pregnancy [12]. Retention of the excessive weight gained during pregnancy (i.e. PPWR) can result in obesity and an increased risk of chronic disease in later life [13–17]. Additionally, maternal obesity negatively affects the health of the offspring in childhood and in adulthood, by increasing the risk of obesity and the related risks of non-communicable diseases. Maternal obesity might therefore result in a vicious circle of obesity throughout generations [4, 18, 19].

Several lifestyle interventions have been implemented during pregnancy in an attempt to reduce GWG and prevent pregnancy- and birth related complications. Although these interventions showed moderate beneficial effects on GWG, they had no significant impact on pregnancy- and birth related complications [20], potentially due to the limited time window [21, 22]. Therefore, the international community of health experts has called for strategies that already intervene in the pre-conception period in order to timely acquire a healthy lifestyle and weight loss [1]. However, such strategies are still scarce [20], possibly due to unpredictability of becoming pregnant and not being linked to the health care system preconceptionally [23]. The few existing studies, though, show promising results in intervening during the preconception period [24–27]. An opportunity to overcome the barrier of reaching women in their preconception period, is to commence interventions already in the postpartum period and continuing until the next pregnancy. Such interpregnancy interventions could potentially be a unique strategy to acquire a healthy lifestyle before a subsequent pregnancy starts.

Effective lifestyle interventions for weight management among postpartum women are usually based on a combination of diet and physical activity [28–30]. Ideally, such lifestyle interventions go beyond education or advice alone and integrate behavioural change techniques such as goal setting, self-monitoring and feedback [25, 31]. Mobile applications are excellent tools to incorporate such behavioural change techniques for intervention efforts promoting a healthy lifestyle [32] that have been found effective in health behaviour change and weight loss [33].

The *inter*pregn*A*ncy *C*oaching for a healthy fu*T*ure (INTER-ACT) intervention is an e-health driven and face-to-face combined coaching program that is implemented between two pregnancies and during the subsequent pregnancy of women with an excessive GWG in the previous pregnancy. The main aim of this study is to assess the effectiveness of the INTER-ACT intervention on the composite outcomes score (GDM, PIH, CS, LGA) in the next pregnancy. This will be evaluated through a randomised controlled trial with an intervention- and control arm.

## Methods

### Study setting

INTER-ACT is a multi-centre randomised controlled trial in which six hospitals from three provinces (Leuven, Antwerp and Limburg) in the Flanders region of Belgium are involved: University Hospital Leuven, University Hospital Antwerp, GasthuisZusters Hospitals Antwerp, St-Franciscus Hospital in Heusden-Zolder, Jessa Hospital in Hasselt, and Hospital Oost-Limburg in Genk. These university, regional, or peripheral hospitals deliver each year between 900 and 2600 new-borns. Recruitment of participants takes place in these hospitals. Baseline measurements, the intervention, and follow-up measurements take place in the hospitals, private clinics, ‘Kind & Gezin’ (Child & Family) organisations, or at home, depending on the preference of the participant.

### Recruitment and eligibility criteria

Study midwives from the participating hospitals are responsible for the recruitment of participants at day 2 or 3 postpartum. To optimise recruitment, flyers and posters are placed in the waiting rooms of the participating hospitals in order to inform potential participants.

Inclusion criteria for participation in the study are the following: women aged ≥18 years; excessive gestational weight gain (above the IOM recommendations [5]) in the previous pregnancy; wish for a next pregnancy not excluded; proficiency of Dutch language; being able to use a smartphone. Exclusion criteria for participation are the following: unable or unwilling to give informed consent; no access to internet; requirement for complex medical diets; history of- or planned bariatric surgery; chronic disorders (e.g. diabetes mellitus type 1 or 2, thyroid disease, renal disease); significant psychiatric disorder; previous stillbirth. Women with twin pregnancies in either the pregnancy preceding the intervention or the subsequent pregnancy are excluded from the study. Participants can not follow other lifestyle interventions during their participation in the INTER-ACT study.

### Randomisation

After inclusion at day 2 or 3 postpartum, an electronic data capture system (CASTOR) will randomise participants in the intervention or control group. Randomisation is revealed at week 6 postpartum. Due to the nature of the intervention (i.e. coaching sessions and use of mobile app, weighing scale and activity tracker), no blinding is involved in this study.

### Intervention

The intervention consists of two intervention phases. The first intervention phase starts at 6 weeks postpartum and lasts until 6 months postpartum. The second intervention phase starts before the 15^th^ week of the next pregnancy and lasts until the 35^th^ week of the pregnancy. A deviation of 2 weeks before or after the planned time point is allowed. Both intervention phases comprise face-to-face coaching and use of a mobile application. Between the two intervention phases, participants in the intervention group receive motivational reminders by e-mail every 3 months.

#### Face-to-face coaching

**Fig. 1:**
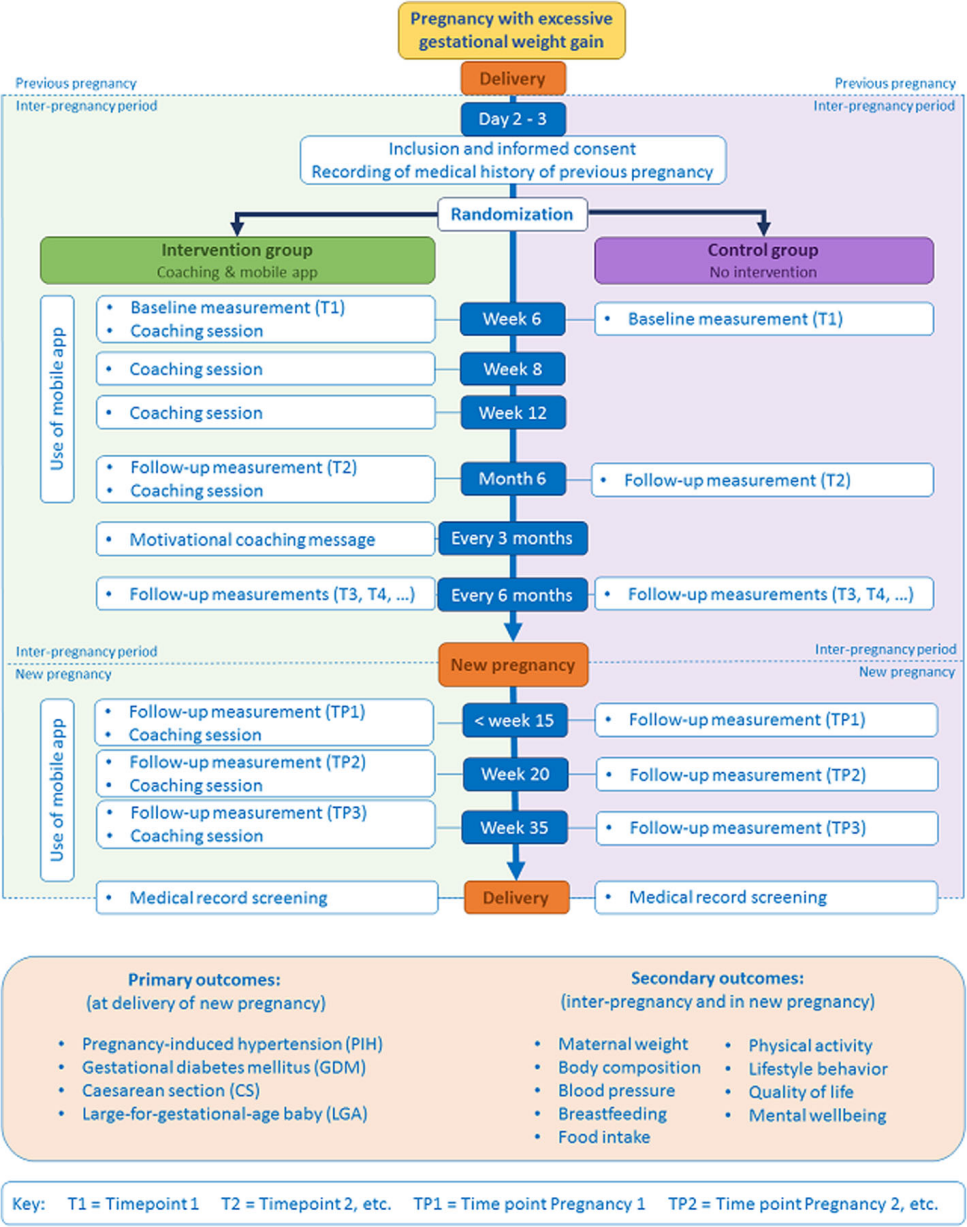
Study design of the INTER-ACT randomised controlled trialᅟ

During both intervention periods, women receive face-to-face coaching sessions: four during the interpregnancy period and three during the next pregnancy (Fig. 1). Coaches trained in motivational interviewing and behavioural change techniques conduct the coaching sessions. During the coaching sessions, the participant is sensitised about the benefits of a healthy lifestyle and the adoption of a healthy lifestyle is stimulated. SMART (Specific, Measurable, Achievable, Relevant and Time specific) goal setting, action planning and reinforcement support the adoption of a healthy lifestyle. Besides, potential barriers to achieving goals or a healthy lifestyle are identified as well as individually tailored solutions to overcome these barriers. The data from the mobile app, i.e. the evolution of body weight, physical activity and mental well-being, support these coaching sessions.

#### Mobile application

The mobile app runs throughout the intervention periods and consists of four domains: nutrition, physical activity, weight, and mental wellbeing. In the nutrition domain, the participant sets nutrition goals based on the Belgian ‘active food triangle’ [34]. If applicable, the nutrition goals are adapted for lactating or pregnant women. On a daily basis, the participant can indicate whether or not she achieved her nutrition goal. In the physical activity domain, the participant sets a goal regarding the number of steps she wants to achieve every day. A Bluetooth-connected activity and sleep tracker (Withings GO™) registers the participant’s activity and sleep, and allows the app to assess whether the physical activity goal is reached. Weight is recorded by a Bluetooth-connected weighing scale (Body Cardio Withings™). In the mental wellbeing domain, the participant can indicate her mood by choosing one out of five emoticons that express different mood states. Besides, the participant can indicate her stress level on a stress meter in the shape of a thermometer. Custom made tips are sent to support the participant in achieving her nutrition and physical activity goals. Additionally, based on the participant’s progress and mood state, the app sends positive coaching messages in order to further motivate the participant. The app development and pilot study of the app including results from evaluation questionnaires will be described in a subsequent paper.

### Ensuring adherence

Adherence to the intervention is enhanced in several ways. Firstly, the coaching sessions coincide with the postnatal follow-up visit and government vaccine scheme (in the interpregnancy phase) and routine ultrasound scans during the 1^st^, 2^nd^ and 3^rd^ trimester of pregnancy (in the new pregnancy phase) in order to reduce barriers to attend the sessions. Secondly, the participants can choose the study location of their preference: at the hospital, private clinic, ‘Child & Family’ organisation, or at the home of the participant. Thirdly, between the two intervention phases, adherence to physical activity and healthy nutrition behaviour are stimulated by sending 3-monthly motivational coaching messages to the participant.

### Participant timeline

Women are recruited at day 2 or 3 postpartum. Coaching sessions take place at week 6, week 8, week 12 and month 6 postpartum. Subsequently, coaching sessions take place before week 15, week 20 and week 35 in the next pregnancy. The mobile app is used from week 6 to month 6 postpartum and in the next pregnancy (Fig. 1).

### Outcomes

In this RCT, we aim to evaluate a composite outcome as primary outcome, of which at least one of the following outcomes occurs in the subsequent pregnancy:

- Pregnancy-induced hypertension (PIH): new-onset elevations of blood pressure (systolic blood pressure > 140 mmHg and diastolic blood pressure >90 mmHg) after 20 weeks of gestation without significant proteinuria [35].
- Gestational diabetes mellitus (GDM): is defined as any degree of glucose intolerance with onset or first recognition during pregnancy [36]. GDM is diagnosed at 24–28 weeks of gestation with the two steps screening strategy which consists of a 50 g glucose challenge test (GCT) and a 2-h 75 g oral glucose tolerance test (OGTT). GDM is diagnosed upon an abnormal GCT (≥ 140 mg/dl) followed by an abnormal OGTT (≥ 153 mg/dl) based on the VDV-VVOG-Domus Medica consensus 2012 and IADPSG criteria [37].
- Caesarean section (CS): surgical procedure in which a foetus is delivered through an incision in the mother’s abdomen and uterus.
- Large-for-gestational-age baby (LGA): birth weight >90^th^ percentile on Flemish sex- and parity-adjusted growth charts [38].

Secondary outcomes are the following:

- Maternal weight
- Body composition
- Blood pressure
- Breastfeeding
- Food intake
- Physical activity
- Lifestyle behaviour
- Quality of life
- Mental wellbeing

### Sample size

The primary endpoint is the composite endpoint of selected pregnancy- and birth-related complications: PIH, GDM, CS and LGA. PIH and GDM are assessed during pregnancy; CS and LGA are determined at time of delivery (Fig. 1). In order to find a significant difference between the intervention and control arm in the rate of the selected pregnancy- and birth-related complications (composite endpoint), assuming a 42% complication rate in the intervention arm and 30% in the control arm (1/4 relative reduction), with a statistical power of 80% and significance level of 0.05, we need 500 women with a next (second) delivery: 250 women in the intervention arm and 250 women in the control arm.

The figures for mean duration between birth and start of a new pregnancy vary around 18 to 24 months. We assume that 2/3 of the included women have a next delivery within 3 years since inclusion in the trial. We also take into account a 30% drop-out rate during follow-up till end of the next pregnancy. In order to obtain 500 women with a next delivery, taking into account a 30% drop-out and only 65% having a next pregnancy within 3 years, we need to include 500x(1/0.65)x(1/0.7) = 1100 women: 550 women per arm.

### Data collection

#### Time of data collection

Clinical data (i.e. medical record data and anthropometric data) and non-clinical data (i.e. data from the mobile app and self-administered questionnaires) are collected at day 2–3, week 6, and month 6 postpartum, every subsequent 6 months until the next pregnancy, and at week <15, 20, and 35 during the next pregnancy (Fig. 1). Deviation of 2 weeks before or after the planned time point is allowed.

At inclusion (day 2 or 3 postpartum) and at the end of the subsequent pregnancy, data will be collected from the medical record. During each coaching session, data from the mobile app will be collected (intervention group only). During all measurement moments (except day 2/3 and delivery of the next pregnancy) anthropometric data and self-administered questionnaires are collected (Fig. 1).

#### Medical record data

Data from the medical record comprise pre-pregnancy weight, pregnancy weight gain, pregnancy- and birth outcomes, data of the new-born such as birthweight and LGA, familial medical history (familial type 2 diabetes mellitus, familial hypertension), chronic disease, psychological history; comorbidity; use of medication; medical history of (previous) pregnancy (GCT, OGTT, GDM, PIH, CS, proteinuria (>300 mg/24 h), preeclampsia, preterm delivery (<37 weeks of gestation) and miscarriage).

#### Anthropometric data

The anthropometric data consist of maternal weight, height, skinfold thickness, waist and hip circumference, body composition, and blood pressure. Maternal height will be measured by a Seca-213 Leicester stadiometer. Maternal weight and body composition (fat mass, fat free mass, muscle mass, extra-cellular water, intra-cellular water, organ fat and phase angle) will be measured with the Tanita MC 780 SMA bio-electric impedance analysis device. Skinfold thickness of the subscapular, suprailiac, biceps, and triceps will be measured with the Harpenden skinfold calliper and evaluated with the Harpenden skinfold calliper software program. Waist and hip circumferences are measured with a Seca 201 measuring tape in order to estimate abdominal body fat. Blood pressure is measured using the Microlife BP A150 AFIB device. All measurements will be performed according to the standard operating procedures to ensure data quality.

#### Mobile app data

Data from the mobile app, i.e. self-monitored weight, physical activity, emotional status, and stress level will be transferred to the secured INTER-ACT website where coaches can retrieve relevant data such as the self-monitored weight and the amount of physical activity.

#### Questionnaires data

A link to the self-administered questionnaires will be sent by e-mail or text message a few days before the study visit so that the participant can complete the questionnaires online before the study visit. Uncompleted questionnaires can be (further) completed during the study visit.

**Socio-demographics questionnaire:** assesses ethnicity, marital status, level of education and employment status.

**Breastfeeding questionnaire**: is based on existing questionnaires by Guelinckx et al. [39] and Bogaerts et al. (unpublished) and assesses type of infant feeding (i.e. breastfeeding, bottle-feeding or a combination), number of feedings per day, duration of having given/giving breastfeeding in weeks, and motives for cessation of breastfeeding.

**Food Frequency Questionnaire:** is developed and validated by Matthys et al. [40] and evaluates on the basis of 25 food items frequency of food intake (per day, per week or per month) and portion size (in gram or millilitre).

**Kaiser Physical Activity Survey (KPAS):** is validated for both pregnant and non-pregnant populations [41, 42] and assesses multiple domains of physical activity (household/caregiving, occupational, active living and sports/exercise).

**Lifestyle behaviour questionnaire:** is based on the questionnaires of the DALI study [43] and evaluates smoking behaviour, alcohol use, sleep duration and quality of sleep, and following a specific diet.

**Short form State Trait Anxiety Inventory six item (sSTAI-6):** measures anxiety symptoms, is validated to the original State Trait Anxiety Inventory (STAI) questionnaire [44] and is reliable and valid for use in the perinatal period [45].

**Edinburgh Postnatal Depression Scale (EPDS):** is a reliable and valid 10-item questionnaire that screens women for symptoms of emotional distress during pregnancy and the postnatal period [46–48].

**Sense of Coherence (SOC):** is measured with the 13-item SOC questionnaire. The SOC-13 assesses comprehensibility, manageability, and meaningfulness of one’s life [49, 50]. It is a valid instrument used for non-pregnant and pregnant populations [51].

**Linear Analog Scale (LAS)**: assesses quality of life by a vertically oriented scale with the lowest score 0, representing a poor quality of life, and the highest score 100 which represent a good quality of life [52].

**Process evaluation questionnaire:** is administered at the end of both intervention periods and evaluates the usability of the mobile app by the System Usability Scale [53], the participants’ experience with the app (i.e. the content of the app) and the face-to-face coaching.

### Safety parameters

Possible adverse events associated with the intervention can be exercise-related adverse events or adverse events related to a rapid weight loss over a short period of time. Moreover, although the EPDS questionnaire is a screening tool and not a diagnostic tool for depression, a positive response on question 10 of the EPDS questionnaire, i.e. having suicidal thoughts, is considered alarming and will be reported to the principal investigator (PI). Based on the result and clinical judgment by the PI, women will be referred to a specialised health practitioner.

### Data management

The data from the questionnaires, the anthropometric data and medical record will be entered and stored in a full Good Clinical Practice (GCP) compliant Electronic Data Capture system, i.e. the CASTOR electronic case report form (eCRF). The data of the mobile application (i.e. physical activity from the activity tracker, weight evolution and mood status) of the participants can be retrieved from the secured website of INTER-ACT, of which only the research team and the participant can have access. Length and weight after delivery will be transferred from the eCRF to the mobile application in order to be able to calculate BMI and show weight curves. In case of no database access, data will be entered on paper CRF and subsequently entered into the data system when access is possible.

### Statistics

All analyses will be carried out using the intention-to-treat principle with data from all participants enrolled in the study. The statistical software SAS version 9.4 will be used. Descriptive statistics for baseline values in the two arms will be presented. There will be no tests of statistical significance or confidence intervals for differences between the two arms, as these are randomised groups. The drop-out rate will be assessed and compared between the two arms. The composite endpoint consists of occurrence of at least one of the following four major pregnancy and birth related complications, at time of next pregnancy: PIH, GDM, CS, and LGA. The rate of the composite endpoint will be calculated and 95% confidence intervals provided in intervention and control arm: 1) in all included patients (intent-to-treat), 2) in all patients completed a next pregnancy. If drop-out rates, reason for drop-out, next pregnancy rates differ between the intervention arm and control arm, the rate of the composite endpoint in patients with a next pregnancy has to be interpreted carefully.

A full statistical analysis plan will be written by the trial statistician prior to any analysis being undertaken. We will report data in line with the Consolidated Standards of Reporting Trials (CONSORT) 2010 Statement [54] and a *P*-values <0.05 will be considered statistically significant.

## Discussion

Excessive weight before and during pregnancy is a public health threat since it may lead to pregnancy- and birth related complications such as pregnancy-induced hypertension, gestational diabetes mellitus, caesarean section, and large for gestational age infants in the short term and weight-related chronic diseases in the long run. Moreover, maternal obesity may lead to inter-generational cycles of obesity through intra-uterine programming of the foetus. Therefore it is essential to timely implement lifestyle interventions targeting high-risk groups.

INTER-ACT is a unique lifestyle intervention that focuses on weight, diet, physical activity and mental well-being between pregnancies and during a subsequent pregnancy with the aim to reduce pregnancy- and birth related complications. The intervention especially focuses on those who are most at risk of these complications in a subsequent pregnancy: women with excessive gestational weight gain during their previous pregnancy.

Strengths of this study can be found in 1) the design of this e-health driven randomised controlled trial, 2) the six participating study sites that represent the northern population of Belgium, and 3) the large sample size calculated as such to demonstrate differences between intervention and control group. A potential pitfall of this study is the long follow-up, i.e. until the end of the subsequent pregnancy, which might result in high drop-out rates. However, possible drop-outs were considered in the power calculations for the needed sample size. This study therefore has the strong potential to show the effectiveness of the e-health driven coaching program for women between and during pregnancies to obtain a healthy lifestyle, to achieve a healthy weight, and to reduce pregnancy- and birth related complications.

## Abbreviations

BMI: Body mass index
CONSORT: Consolidated Standards of Reporting Trials
CS: Caesarean section
eCRF: Electronic case report form
EGWG: Excessive gestational weight gain
EPDS: Edinburgh Postnatal Depression Scale
FWO: Fund for Scientific Research
GCP: Good clinical practice
GCT: Glucose challenge test
GDM: Gestational diabetes mellitus
GWG: Gestational weight gain
IOM: Institute of Medicine
KPAS: Kaiser Physical Activity Survey
LAS: Linear Analog Scale
LGA: Large-for-gestational-age
OGTT: Oral glucose tolerance test
PIH: Pregnancy-induced hypertension
PPWR: Postpartum weight retention
SMART: Specific, measurable, achievable, relevant and time specific
SOC: Sense of Coherence
sSTAI-6: Short form State Trait Anxiety Inventory six item

## References

[1] Poston L, Caleyachetty R, Cnattingius S, Corvalán C, Uauy R, Herring S (2016). Preconceptional and maternal obesity: epidemiology and health consequences. Lancet Diabetes Endocrinol.

[2] Poobalan A, Aucott L, Gurung T, Smith WCS, Bhattacharya S (2009). Obesity as an independent risk factor for elective and emergency caesarean delivery in nulliparous women–systematic review and meta‐analysis of cohort studies. Obes Rev.

[3] Chu SY, Callaghan WM, Kim SY, Schmid CH, Lau J, England LJ (2007). Maternal obesity and risk of gestational diabetes mellitus. Diabetes Care.

[4] Catalano P, Ehrenberg H (2006). Review article: The short‐and long‐term implications of maternal obesity on the mother and her offspring. BJOG.

[5] Rasmussen KM, Yaktine AL. Weight gain during pregnancy: reexamining the guidelines. Washington, DC: National Academies Press; 2010. 20669500

[6] Hedderson MM, Gunderson EP, Ferrara A (2010). Gestational Weight Gain and Risk of Gestational Diabetes Mellitus. Obstet Gynecol.

[7] Chung JG, Taylor RS, Thompson JM, Anderson NH, Dekker GA, Kenny LC (2013). Gestational weight gain and adverse pregnancy outcomes in a nulliparous cohort. Eur J Obstet Gynecol Reprod Biol.

[8] Ferraro ZM, Barrowman N, Prud’homme D, Walker M, Wen SW, Rodger M (2012). Excessive gestational weight gain predicts large for gestational age neonates independent of maternal body mass index. J Matern Fetal Neonatal Med.

[9] Haugen M, Brantsæter AL, Winkvist A, Lissner L, Alexander J, Oftedal B (2014). Associations of pre-pregnancy body mass index and gestational weight gain with pregnancy outcome and postpartum weight retention: a prospective observational cohort study. BMC Pregnancy Childbirth.

[10] Xiong C, Zhou A, Cao Z, Zhang Y, Qiu L, Yao C (2016). Association of pre-pregnancy body mass index, gestational weight gain with cesarean section in term deliveries of China. Scientific Reports.

[11] Bogaerts A, Van den Bergh B, Nuyts E, Martens E, Witters I, Devlieger R (2012). Socio-demographic and obstetrical correlates of pre-pregnancy body mass index and gestational weight gain. Clin Obes.

[12] Bogaerts A, Van den Bergh BR, Ameye L, Witters I, Martens E, Timmerman D (2013). Interpregnancy weight change and risk for adverse perinatal outcome. Obstet Gynecol.

[13] Endres LK, Straub H, McKinney C, Plunkett B, Minkovitz CS, Schetter CD (2015). Postpartum Weight Retention Risk Factors and Relationship to Obesity at One Year. Obstet Gynecol.

[14] Rooney BL, Schauberger CW, Mathiason MA (2005). Impact of perinatal weight change on long-term obesity and obesity-related illnesses. Obstet Gynecol.

[15] Kirkegaard H, Stovring H, Rasmussen KM, Abrams B, Sorensen TI, Nohr EA (2014). How do pregnancy-related weight changes and breastfeeding relate to maternal weight and BMI-adjusted waist circumference 7 y after delivery? Results from a path analysis. Am J Clin Nutr.

[16] Mongraw-Chaffin ML, Anderson CA, Clark JM, Bennett WL (2014). Prepregnancy body mass index and cardiovascular disease mortality: the Child Health and Development Studies. Obesity (Silver Spring).

[17] Liu H, Zhang C, Zhang S, Wang L, Leng J, Liu D (2014). Prepregnancy body mass index and weight change on postpartum diabetes risk among gestational diabetes women. Obesity (Silver Spring).

[18] Troesch B, Biesalski HK, Bos R, Buskens E, Calder PC, Saris WH (2015). Increased intake of foods with high nutrient density can help to break the intergenerational cycle of malnutrition and obesity. Nutrients.

[19] Ozanne SE (2015). Epigenetic signatures of obesity. N Engl J Med.

[20] Hanson M, Barker M, Dodd JM, Kumanyika S, Norris S, Steegers E, et al. Interventions to prevent maternal obesity before conception, during pregnancy, and post partum. Lancet Diabetes Endocrinol. 2016;5(1):65–76. 10.1016/S2213-8587(16)30108-5 27743974

[21] Dodd JM, Turnbull D, McPhee AJ, Deussen AR, Grivell RM, Yelland LN (2014). Antenatal lifestyle advice for women who are overweight or obese: LIMIT randomised trial. BMJ.

[22] Poston L, Bell R, Croker H, Flynn AC, Godfrey KM, Goff L (2015). Effect of a behavioural intervention in obese pregnant women (the UPBEAT study): a multicentre, randomised controlled trial. Lancet Diabetes Endocrinol.

[23] Barker M, Baird J, Lawrence W, Vogel C, Stömmer S, Rose T (2016). Preconception and pregnancy: opportunities to intervene to improve women’s diets and lifestyles. J Dev Orig Health Dis.

[24] Hadar E, Ashwal E, Hod M (2015). The preconceptional period as an opportunity for prediction and prevention of noncommunicable disease. Best Pract. Res. Clin. Obstet. Gynaecol..

[25] Witkop CT. Preconception and Pregnancy Care in Overweight or Obese Woman. In: Obesity During Pregnancy in Clinical Practice. London: Springer; 2014. p. 33–52.

[26] Dean SV, Lassi ZS, Imam AM, Bhutta ZA (2014). Preconception care: nutritional risks and interventions. Reprod Health.

[27] Haire-Joshu D, Tabak R (2016). Preventing Obesity Across Generations: Evidence for Early Life Intervention. Annu Rev Public Health.

[28] Choi J, Fukuoka Y, Lee JH (2013). The effects of physical activity and physical activity plus diet interventions on body weight in overweight or obese women who are pregnant or in postpartum: a systematic review and meta-analysis of randomized controlled trials. Prev Med.

[29] Lim S, O’Reilly S, Behrens H, Skinner T, Ellis I, Dunbar J (2015). Effective strategies for weight loss in post‐partum women: a systematic review and meta‐analysis. Obes Rev.

[30] Nascimento S, Pudwell J, Surita F, Adamo K, Smith G (2014). The effect of physical exercise strategies on weight loss in postpartum women: a systematic review and meta-analysis. Int J Obes.

[31] Abraham C, Michie S (2008). A taxonomy of behavior change techniques used in interventions. Health Psychol.

[32] Bardus M, van Beurden SB, Smith JR, Abraham C (2016). A review and content analysis of engagement, functionality, aesthetics, information quality, and change techniques in the most popular commercial apps for weight management. Int J Behav Nutr Phys Act.

[33] Lyzwinski LN (2014). A systematic review and meta-analysis of mobile devices and weight loss with an intervention content analysis. J Personalized Med.

[34] Schildknechtstraat G, Vanhauwaert E. De actieve voedingsdriehoek: een praktische voedings-en beweeggids. 2012.

[35] Obstetricians ACo, Gynecologists. Hypertension in pregnancy. Report of the American college of obstetricians and gynecologists’ task force on hypertension in pregnancy. Obstet Gynecol. 2013;122(5):1122. 10.1097/01.AOG.0000437382.03963.88 24150027

[36] American Diabetes Association (2014). tandards of medical care in diabetes--2014. Diabetes Care.

[37] Benhalima K (2013). The VDV-VVOG-Domus Medica consensus 2012 on screening for pregestational diabetes in pregnancy and screening for gestational diabetes. P Belg Roy Acad Med.

[38] Devlieger H, Martens G, Bekaert A, Eeckels R (2000). Standaarden van geboortegewicht voor zwangerschapsduur voor de Vlaamse boreling. Tijdschr Geneesk.

[39] Guelinckx I, Devlieger R, Bogaerts A, Pauwels S, Vansant G (2012). The effect of pre-pregnancy BMI on intention, initiation and duration of breast-feeding. Public Health Nutr.

[40] Matthys C, Meulemans A, Van Der Schueren B, editors. Development and validation of general FFQ for use in clinical practice. Ann Nutr Metab; 2015: Krager.

[41] Ainsworth BE, Sternfeld B, Richardson MT, Jackson K (2000). Evaluation of the kaiser physical activity survey in women. Med Sci Sports Exerc.

[42] Schmidt MD, Freedson PS, Pekow P, Roberts D, Sternfeld B, Chasan-Taber L (2006). Validation of the Kaiser Physical Activity Survey in pregnant women. Med Sci Sports Exerc.

[43] Jelsma JG, van Poppel MN, Galjaard S, Desoye G, Corcoy R, Devlieger R (2013). DALI: Vitamin D and lifestyle intervention for gestational diabetes mellitus (GDM) prevention: an European multicentre, randomised trial–study protocol. BMC Pregnancy Childbirth.

[44] van der Bij AK, de Weerd S, Cikot RJ, Steegers EA, Braspenning JC (2003). Validation of the dutch short form of the state scale of the Spielberger State-Trait Anxiety Inventory: considerations for usage in screening outcomes. Community Genet.

[45] Bayrampour H, McDonald S, Fung T, Tough S (2014). Reliability and validity of three shortened versions of the State Anxiety Inventory scale during the perinatal period. J Psychosom Obstet Gynaecol.

[46] Murray L, Carothers AD (1990). The validation of the Edinburgh Post-natal Depression Scale on a community sample. Br J Psychiatry.

[47] Bergink V, Kooistra L, Lambregtse-van den Berg MP, Wijnen H, Bunevicius R, van Baar A (2011). Validation of the Edinburgh Depression Scale during pregnancy. J Psychosom Res.

[48] Bogaerts AF, Devlieger R, Nuyts E, Witters I, Gyselaers W, Guelinckx I (2013). Anxiety and depressed mood in obese pregnant women: a prospective controlled cohort study. Obes Facts.

[49] Eriksson M, Lindstrom B (2005). Validity of Antonovsky’s sense of coherence scale: a systematic review. J Epidemiol Community Health.

[50] Antonovsky A (1993). The structure and properties of the sense of coherence scale. Soc Sci Med.

[51] Linden K, Sparud-Lundin C, Adolfsson A, Berg M (2016). Well-Being and Diabetes Management in Early Pregnant Women with Type 1 Diabetes Mellitus. Int J Environ Res Public Health.

[52] Moons P, Budts W, De Geest S (2006). Critique on the conceptualisation of quality of life: a review and evaluation of different conceptual approaches. Int J Nurs Stud.

[53] Brooke J, Jordan PW, Thomas B, Weerdmeester BA, McClelland AL (1996). SUS: a “quick and dirty” usability scale. Usability Evaluation in Industry.

[54] CONSORT. http://www.consort-statement.org/consort-2010. Accessed 10 Jan 2017.

